# Insight into ECMO, mortality and ARDS: a nationwide analysis of 45,647 ECMO runs

**DOI:** 10.1186/s13054-021-03463-2

**Published:** 2021-01-28

**Authors:** Benjamin Friedrichson, Haitham Mutlak, Kai Zacharowski, Florian Piekarski

**Affiliations:** 1 Department of Anaesthesiology, Intensive Care Medicine and Pain Therapy, University Hospital Frankfurt, Goethe University, Theodor-Stern-Kai 7, 60590 Frankfurt am Main, Germany; 2 Department of Anaesthesiology, Intensive Care Medicine and Pain Therapy, SANA Klinikum Offenbach, Starkenburgring 66, 63069 Offenbach am Main, Germany

**Keywords:** ARDS, Extracorporeal life support, ECMO, OPS, Mortality

## Abstract

**Background:**

Extracorporeal life support (ECLS) has become an integral part of modern intensive therapy. The choice of support mode depends largely on the indication. Patients with respiratory failure are predominantly treated with a venovenous (VV) approach. We hypothesized that mortality in Germany in ECLS therapy did not differ from previously reported literature

**Methods:**

Inpatient data from Germany from 2007 to 2018 provided by the Federal Statistical Office of Germany were analysed. The international statistical classification of diseases and related health problems codes (ICD) and process keys (OPS) for extracorporeal membrane oxygenation (ECMO) types, acute respiratory distress syndrome (ARDS) and hospital mortality were used.

**Results:**

In total, 45,647 hospitalized patients treated with ECLS were analysed. In Germany, 231 hospitals provided ECLS therapy, with a median of 4 VV-ECMO and 9 VA-ECMO in 2018. Overall hospital mortality remained higher than predicted in comparison to the values reported in the literature. The number of VV-ECMO cases increased by 236% from 825 in 2007 to 2768 in 2018. ARDS was the main indication for VV-ECMO in only 33% of the patients in the past, but that proportion increased to 60% in 2018. VA-ECMO support is of minor importance in the treatment of ARDS in Germany. The age distribution of patients undergoing ECLS has shifted towards an older population. In 2018, the hospital mortality decreased in VV-ECMO patients and VV-ECMO patients with ARDS to 53.9% (*n* = 1493) and 54.4% (*n* = 926), respectively.

**Conclusions:**

ARDS is a severe disease with a high mortality rate despite ECLS therapy. Although endpoints and timing of the evaluations differed from those of the CESAR and EOLIA studies and the Extracorporeal Life Support Organization (ELSO) Registry, the reported mortality in these studies was lower than in the present analysis. Further prospective analyses are necessary to evaluate outcomes in ECMO therapy at the centre volume level.

## Introduction

Extracorporeal life support (ECLS) has become an integral part of modern intensive therapy. Various pulmonary and cardiac conditions are indications for the use of ECLS [1]. Different terms are used in the literature for ECLS depending on the system and type of cannulation. This article divides ECLS as recommended by The Extracorporeal Life Support Organization Maastricht Treaty into pump-operated extracorporeal membrane oxygenation (ECMO) systems and pumpless extracorporeal carbon dioxide removal (ECCO_2_R) systems [2]. ECMO is further classified into venoarterial-ECMO (VA-ECMO) and venovenous-ECMO (VV-ECMO), depending on the support mode. The choice of support mode depends largely on the indication. Patients who have undergone lung replacement due to respiratory failure are mainly treated with VV-ECMO, but the cannulation strategy is often venoarterial (VA) or venovenoarterial (VVA) for patients with combined cardiac and pulmonary failure. VA cannulation is used for cardiac support and is useful for cardiac and cardiosurgical indications as well as for extracorporeal cardiopulmonary resuscitation (E-CPR).

The main indication for the use of VV-ECMO is ARDS. Over the past decades, increasing implantation rates and decreasing mortality rates in patients with ARDS of different aetiologies have been observed [3]. In contrast, Karagiannidis et al. 2016 showed the opposite results in a retrospective analysis of all ECLS therapies in the German health care system [4].

The Federal Statistical Office has a uniform and valid collection of data covering all of Germany that is recorded via the diagnosis-related group (DRG) system. In cooperation with the Federal Statistical Office, the following questions were examined. The quality of the register data has already been evaluated by our research team in projects on healthcare system data and transfusion data [5, 6].

We hypothesized that the mortality in Germany in ECMO therapy in ARDS does not differ from previously reported literature. For this purpose, routine data from the German Federal Statistical Office on ECLS treatment and their results regarding mortality, treatment duration and age distribution in ARDS patients from the entire inpatient treatments were evaluated.

## Materials and methods

### Data source and population

Data were provided by the Federal Statistical Office of Germany. Since the register data are anonymous, no ethics approval was necessary. Hospitals in Germany are obliged by law to report diagnoses according to the international statistical classification of diseases and related health problems (ICD) codes and process keys (OPS). Data from every German clinic are collected in a structured way, ensuring that highly representative data are available [7]. The data were requested from the Federal Statistical Office for scientific use and publication.

The registry was screened for ECLS therapy and ARDS-related ECLS therapy as defined by the ICD and OPS. ARDS was coded as ICD J80, VV-ECMO as OPS 8-852.0X, and VA-ECMO as 8-852.3X. It is possible to limit the combination of data using a special query. We queried the combination of ARDS (J80), discharged patients, in-hospital death and the respective ECMO therapies. To record all patients suffering from ARDS, those with a main or secondary diagnosis of ARDS were selected [8].

All age groups and data from 2007 to 2018 were included. More recent data were not available due to accounting aspects and the internal data validation processes of the Federal Statistical Office. At the beginning of the New Year, the hospitals transmit the data from the previous calendar year to the Institute for the Hospital Remuneration System (InEK), at which point the data are forwarded to the Federal Statistical Office. The Federal Statistical Office processes and checks the data for validity and releases them for further scientific analyses. This process usually requires a period of approximately 1.5 years. Intraoperative ECMO therapies were excluded from the analysis due to differences in coding.

### Statistical analysis and outcome

The data were analysed descriptively. The absolute numbers were stratified by age groups and years. The mortality rate was stratified by the different ECMO modes and annual duration of treatment. The annual incidences were calculated as described above. Excel for Mac (Release 16.37, Microsoft Corp., Seattle, WA, USA), SPSS (Release 22, IBM SPSS, Armond, NY, USA) and Prism 8 for MacOS (Release 8.4.3, GraphPad Software, San Diego, CA, USA) were used for the analyses.

## Results

**Fig. 1:**
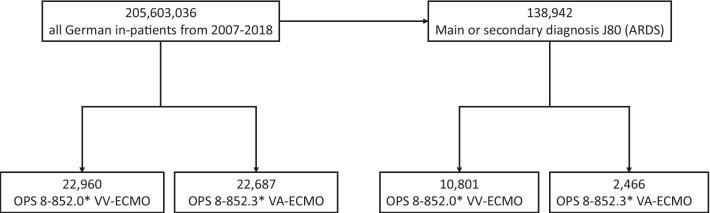
Patient flow chart

In total, 45,647 hospitalized patients treated with ECLS were analysed from 2007 to 2018 (Table 1 and Fig. 1).

**Table 1:** Patient characteristics of all patients treated with ECLS in Germany from 2007 to 2018

	All patients		ARDS	
	VA-ECMO	VV-ECMO	VA-ECMO	VV-ECMO
Total, *n*	22,687	22,960	2,466	10,801
Female, *n* (%)	6873 (30,3)	7731 (33,7)	–	–
Nonsurvivor, *n*	14,729	13,495	1,691	6,155
Mortality, %	65.6	53.9	69.6	54.4
Age groups (years)				
Under 1	1340 (5.4)	1249 (5.4)	–	–
1–5, *n* (%)	170 (0.7)	158 (0.7)	–	–
5–10, *n* (%)	95 (0.4)	95 (0.4)	–	–
10–15, * n* (%)	147 (0.6)	143 (0.6)	–	–
15–20, * n* (%)	413 (1.7)	396 (1.7)	–	–
20–25, * n* (%)	593 (2.4)	614 (2.7)	–	–
25–30, * n* (%)	636 (2.6)	663 (2.9)	–	–
30–35, * n* (%)	765 (3.1)	761 (3.3)	–	–
35–40, * n* (%)	957 (3.9)	960 (4.2)	–	–
40–45, * n* (%)	1207 (4.9)	1195 (5.2)	–	–
45–50, * n* (%)	2003 (8.1)	1970 (8.6)	–	–
50–55, * n* (%)	2542 (10.3)	2472 (10.8)	–	–
55–60, * n* (%)	3056 (12.4)	2919 (12.7)	–	–
60–65, * n* (%)	2956 (11.9)	2766 (12)	–	–
65–70, * n* (%)	2699 (10.9)	2475 (10.8)	–	–
70–75,* n* (%)	2453 (9.9)	2202 (9.6)	–	–
75–80,* n* (%)	1700 (6.9)	1427 (6.2)	–	–
80–85,* n* (%)	516 (2.1)	392 (1.7)	–	–
85–90,* n* (%)	125 (0.5)	99 (0.4)	–	–
90–95,* n* (%)	9 (0)	4 (0)	–	–
95 and older, *n* (%)	1 (0)	0 (0)	–	–

In Germany, 231 hospitals provided ECLS therapy in 2018 (Table 2). Of these, 173 hospitals provided VA-ECMO and 231 VV-ECMO. The annual case volume was low, with a median of 4 for VV-ECMO and 9 for VA-ECMO.

**Table 2:** Hospitals providing ECLS support in Germany in 2018

	ECMO centre (*n*)	
	VV-ECMO	VA-ECMO
Median (Q1;Q3)	4 (1;12)	9 (2;31)
Total centres	231	173
*Annual ECMO runs/centre*		
0–10	168	93
11–20	25	21
21–30	15	13
31–40	5	10
41–50	5	7
50 and more	13	29

### Patient characteristics

See Table 1.

### ECMO centres in Germany 2018

See Table 2.

### Venovenous-ECMO

**Fig. 2:**
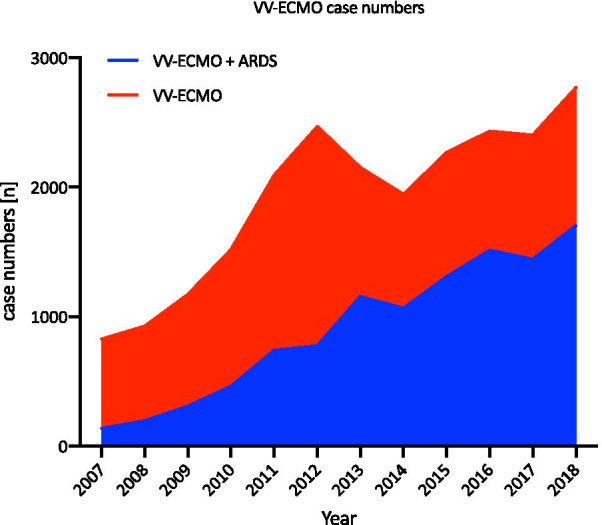
VV-ECMO cases from 2007 to 2018. Case numbers of patients receiving VV-ECMO and patients diagnosed with ARDS an VV-ECMO from 2007 to 2018

**Fig. 3:**
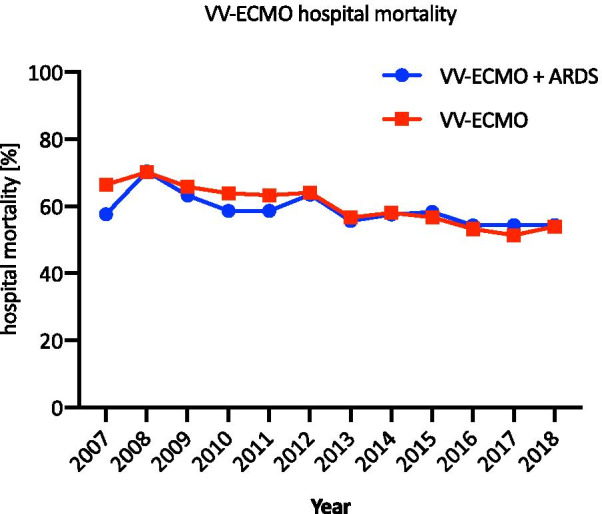
VV-ECMO hospital mortality from 2007 to 2018. The hospital mortality is shown for patients receiving VV-ECMO with and without ARDS from 2007 to 2018

The number of VV-ECMO cases increased by 236% from 825 in 2007 to 2768 in 2018. In 2012, the number of VV-ECMO cases reached an initial peak of 2468 and then decreased by 21.2% (*n* = 524) in the following years leading to 2014. After 2014, there was renewed growth that continued until 2018 (increase of 42.4%, *n* = 824). VV-ECMO therapy in ARDS patients increased by 1143% from 2007 (*n* = 137) to 2018 (*n* = 1703), as shown in Fig. 2. The percentage of patients treated with VV-ECMO who had ARDS increased substantially from 16.6% in 2007 (*n* = 137) to 61.5% in 2018 (*n* = 1703). Hospital mortality was the highest in 2008: 70.1% in all VV-ECMO (*n* = 649) patients and 70.4% in VV-ECMO (*n* = 138) patients with ARDS (shown in Fig. 3). In 2018, the hospital mortality decreased in VV-ECMO patients and VV-ECMO patients with ARDS to 53.9% (*n* = 1493) and 54.4% (*n* = 926), respectively.

**Fig. 4:**
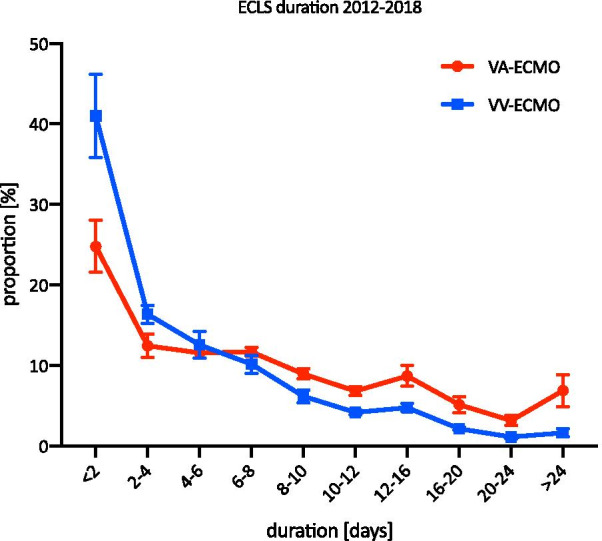
The distribution of treatment duration in ECLS from 2012 to 2018. The mean treatment duration of ECLS therapy from 2012 to 2018 is shown (mean and SD)

**Fig. 5:**
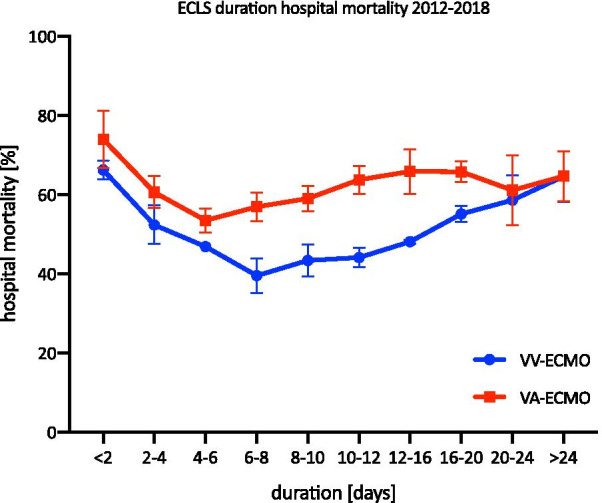
Hospital mortality of ECLS in relation to treatment duration from 2012 to 2018. The mean hospital mortality of ECLS in relation to treatment duration is shown (mean and SD)

The average duration of VV-ECMO use from 2012 to 2018 is shown in Fig. 4. Overall, 24.8% (standard deviation (SD) = 3.2%) of VV-ECMO patients used it for less than 2 days, and 6.9% (SD = 2%) used it for more than 24 days. The hospital mortality was the highest in patients with a treatment length shorter than 2 days (69.7%, SD = 3.4%) and the lowest in those who received 6–8 days of treatment (43%, SD = 5.2%) (Fig. 5). In 2017 and 2018, 0.87% and 1.7% were treated with VV-ECMO for 48 days or longer, respectively.

**Fig. 6:**
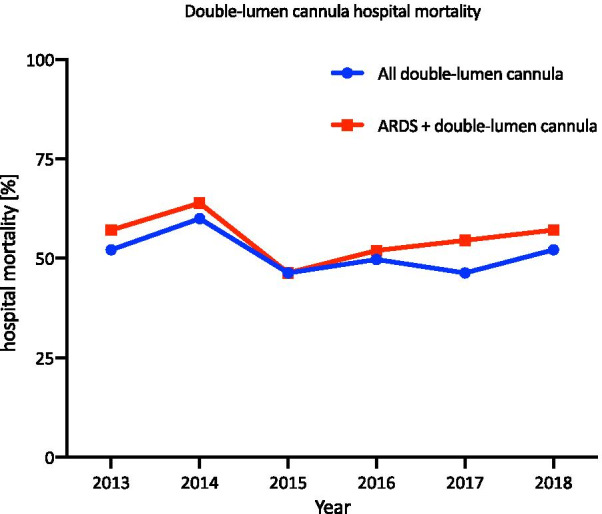
Double-lumen cannula hospital mortality from 2013 to 2018. The hospital mortality for patients treated with a double-lumen cannula and patients diagnosed with ARDS and double-lumen cannula are shown from 2013 to 2018

Since 2013, the use of a double-lumen cannula has been documented by OPS. The use of a double-lumen cannula remained uncommon in all ECLS cases from 2013 to 2018 (3.2%). On average, hospital mortality among all patients with double-lumen cannulas was 51.1% (SD = 4.6%), while in those with double-lumen cannulas and ARDS, the hospital mortality rate was 55.2% (SD = 5.4%) (Fig. 6).

### Venoarterial-ECMO

**Fig. 7:**
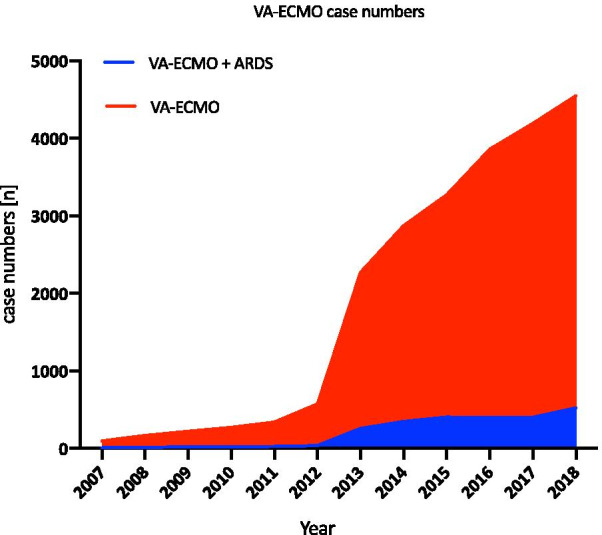
VA-ECMO cases from 2007 to 2018. Case numbers of patients receiving VA-ECMO and patients diagnosed with ARDS and VA-ECMO from 2007 to 2018

**Fig. 8:**
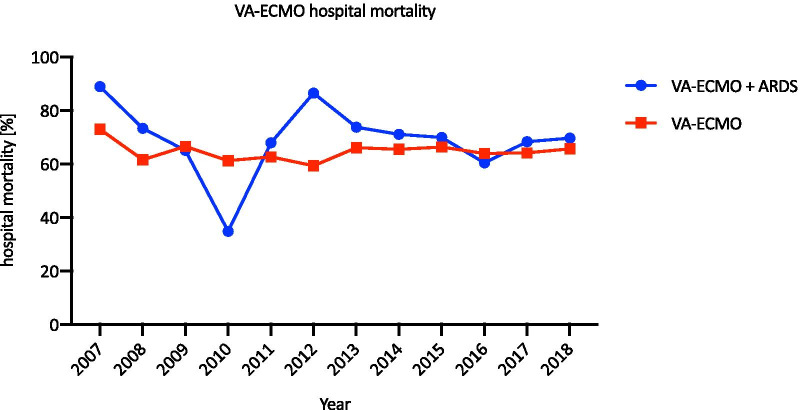
VA-ECMO hospital mortality from 2007 to 2018. The hospital mortality is shown for patients receiving VA-ECMO with and without ARDS from 2007 to 2018

The number of VA-ECMO cases increased by 4639% from 96 in 2007 to 4549 in 2018, with a sharp increase after 2013 (Fig. 7). The ratio of patients with ARDS and VA-ECMO support to all VA-ECMO patients remained low, with a maximum of 12% in all years. In Fig. 8, a variable but high hospital mortality rate of all VA-ECMO patients and VA-ECMO patients with ARDS is shown. The highest mortality rate in all VA-ECMO patients was observed in 2007 (72.9%, *n* = 70); in the same year, the mortality rate in VA-ECMO patients with ARDS was 88.9% (*n* = 8). The lowest mortality in all VA-ECMO cases was seen in 2012 (59.3%, *n* = 341), which increased in 2013 to 66% (*n* = 1,498) and remained at 65.6% (*n* = 2984) until 2018.

The average duration of the use of VA-ECMO from 2012 to 2018 is shown in Fig. 4. 41% (SD = 5.1%) of all VA-ECMO patients used it for less than 2 days, and 1.6% (SD = 0.5%) used it for more than 24 days. Similar to the VV-ECMO group, the hospital mortality was the highest in those with a treatment length less than 2 days (73.9%, SD = 7.3%) and the lowest in those with 4–6 days of treatment (53.4%, SD = 3%). The hospital mortality rate increased to 65.8% (SD = 5.6%) in the group treated for 12–16 days. In 2017 and 2018, 5 and 8 patients, respectively, were treated with VA-ECMO for 48 days or longer.

### Age distribution

**Fig. 9:**
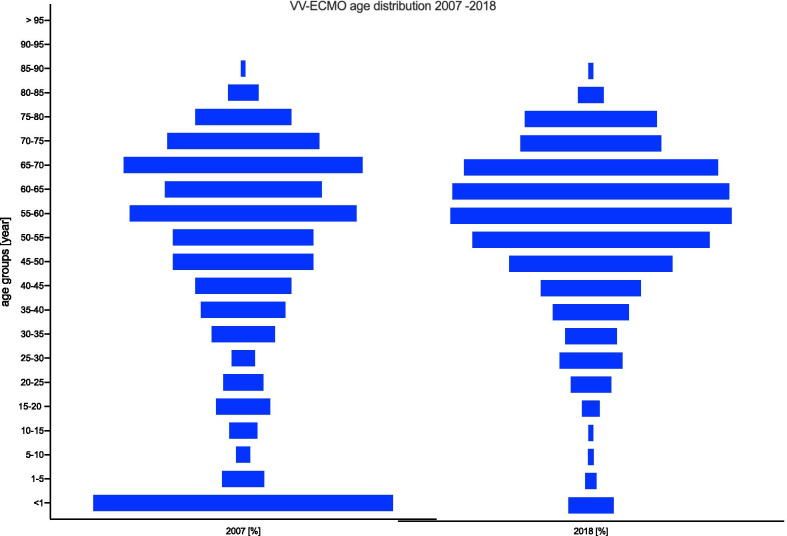
Age distribution of VV-ECMO patients from 2007 to 2018

**Fig. 10:**
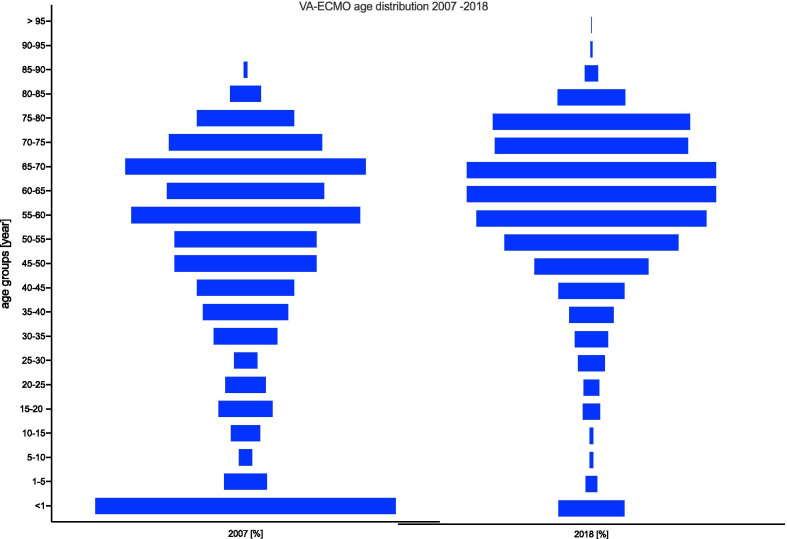
Age distribution of VA-ECMO patients from 2007 to 2018

The age distribution of patients undergoing VV-ECMO changed noticeably from 2007 to 2018, as shown in Fig. 9. In particular, the use in children under one year of age decreased from 15.5% in 2007 to less than 2.3% in 2018. The largest increase occurred in the 60- to 65-year-old age group from 8.1% in 2007 to 14.3% in 2018. A similar change was observed in the age distribution of patients undergoing VA-ECMO from 2007 to 2018 (Fig. 10). The use of VA-ECMO in children under one year of age decreased from 15.5% in 2007 to less than 3.4% in 2018. The two largest increases were observed in the 60- to 65-year-old and 75- to 80-year-old age groups from 2007 to 2018. In 2018, 6 patients older than 90 years were treated with VA-ECMO.

## Discussion

This study presents an analysis of the largest number of cases of ECLS therapy in ARDS patients worldwide.

The main findings of the present analysis can be summarized as follows.

First, in Germany, the overall hospital mortality rate in patients receiving ECLS therapy remains higher than the rates reported in the literature. Second, the median annual centre volume of 4 in VV-ECMO and 9 in VA-ECMO is low. Third, ARDS as the main indication for VV-ECMO was only identified in 33% of patients in the past, although this proportion increased to 60% by 2018. Fourth, the age distribution of ECLS patients has shifted towards an older group.

### Mortality

A comparison of the mortality rates in patients receiving ECMO therapy for ARDS from the ELSO registry with the data in this study revealed a large gap. In 2016, the mortality rate among all ECLS patients with ARDS was 55.6% in Germany, while it was only 45% in adult patients in the ELSO registry [3]. According to the latest data from the ELSO registry, mortality in adult ARDS patients receiving ECLS was only 39% over the period from 2015 to 25.1.2020 [9]. One possible explanation is bias because very few hospitals worldwide submit their data to ELSO, and most of the submitting hospitals are experienced, high-volume centres that actively contribute their data. The median for the annual centre volume from the European ELSO registry in 2019 was 18 cases per centre [9]. In contrast, Germany had a distinctly lower median of 4 cases per centre in 2018. In Germany, only 21 out of 1,160 hospitals with intensive care units are registered with ELSO [10, 11]. In 2018, in the European ELSO Registry, only 81 centres with 2,233 cases were reported. In contrast, in Germany, there were 231 centres with 7,317 cases reported, which shows a high number of cases unreported to the registry. There is no minimum standard for ECLS therapy in Germany by law, and registration in a programme, such as ELSO or the German ARDS network (79 registered hospitals), does not obligate hospitals to implement or adhere to specific standards. This finding highlights the importance of treating patients in specialized centres to provide the survival benefit seen in ELSO centres to all patients. International and national recommendations for the minimum standards for ECLS therapy have existed for a long time, but their implementation has been lacking [12, 13]. The shuttle-spoke model, which was already recommended in 2014 by the International ECMO Network (ECMONet), should also be reconsidered as a possible solution for Germany, in which each federal state establishes a central ECLS centre (hub) and all regional hospitals transfer their patients to the hub [14]. Comparing the hospital mortality rates in 2011 (63%) with data from the US health care system, Sauer et al. showed a similarly high mortality rate of 61% among all ECLS patients [15]. In contrast, the mortality rates in the two largest randomized studies, the CESAR trial and the EOLIA trial, were relatively low for ARDS patients treated with ECLS (37% and 38%) [16, 17]. Again, treatment was mainly administered at specialized centres, and there were strict inclusion criteria, which may have contributed to these mortality rates. Treatment in specialized centres clearly results in reduced mortality. Similarly, Muguruma et al. showed a significantly lower mortality rate in patients treated in high-volume ECMO centres (50.4%) than in those treated in low-volume centres (62.5%) [18]. Therapy with ECMO is a multidisciplinary process and requires a high level of experience for all persons involved. Therefore, locally adapted standard operating procedures with regard to the indication for ECLS, selection of the mode of ECLS, catheterization strategy, therapy parameters and weaning protocols are recommended, and the implementation of these standards, including the introduction of a minimum number of ECLS therapies through state regulations, should be reconsidered given the high mortality rates.

The mortality rates varied substantially with treatment duration. The highest hospital mortality rate (69.7%) was observed in the group treated for less than 2 days; conversely, the lowest rate (43%) was observed in the group treated between 6 and 8 days. This shows that patients who benefit from VV-ECMO therapy need approximately one week before they can be successfully weaned from ECLS with favourable outcomes. In the case of the patients who died after less than 2 days of therapy, it should be considered whether the indication for ECMO therapy was identified too late in these patients, becoming an exclusion criterion for ECLS therapy, or whether the indication for the initiation of ECLS was questionable. A similar pattern was observed in patients receiving VA-ECMO. Again, the highest mortality was in the group that received treatment for less than 2 days, and the lowest mortality rate was in the group treated for 4–6 days (73.9% vs 53.4%). Compared to VV-ECMO, however, patients receiving VA-ECMO had a significantly higher mortality over the treatment period, possibly due to the main indication for VA-ECMO, cardiogenic shock, which is associated with increased mortality.

### Age distribution

Regarding the age distribution, a trend towards older patients was observed for both ECLS procedures. In 2007, 54.2% of all VA-ECMO and VV-ECMO patients were older than 50 years of age, whereas 71.2% of VA-ECMO and 70.1% of VV-ECMO patients in 2018 were older than 50 years. With regard to the age group older than 75 years, the percentage of patients in the VA-ECMO group doubled from 6.8% in 2007 to 14.6% in 2018; in comparison, the age group older than 75 years receiving VV-ECMO increased only slightly. In 2007, 6.8% of the patients receiving VV-ECMO were older than 75 years, and this proportion increased only slightly to 8.4% by 2018. In 2018, 6 patients older than 90 years were treated with ECLS. The extension of ECLS therapy to older patients is the subject of debate based on results published in the literature, which show that older age increases the risk of mortality [19].

### VV-ECMO

In this article, the historical evolution of individual ECLS procedures as therapies for ARDS is shown. The number of patients treated with VV-ECMO has grown substantially, apart from a brief decline in 2012. The decreasing trend from 2012 onwards described by Karagiannidis et al. in 2016 has not continued, and the runs in 2018 clearly exceeded those in 2012 [4]. In the context of the COVID-19 pandemic, the peak of the increase in VV-ECMO treatments has probably not yet been reached. The proportion of VV-ECMO patients with ARDS increased rapidly from 31.4 to 53.7% between 2012 and 2013. What are the reasons for the large increase in ECMO runs since 2009? First, studies showed favourable outcomes in the context of the H1N1 pandemic [20–22]. Second, the results of the CESAR trial 2008 were released, which showed that ECMO led to improved outcomes, although there was some criticism of the trial [17]. Third, there was better availability and easier implementation of ECLS therapy due to the introduction of modern ECLS devices at the end of 2008.

The sharp increase in VV-ECMO patients with ARDS and the almost constant proportion of VV-ECMO patients with ARDS until 2012 may indicate increased use in critically ill patients, possibly without adequate indication of the need for VV-ECMO support. A possible explanation for this is the fact that there are monetary aspects to the DRG system. It is unclear whether the increased number of ECMO centres in Germany has led to a higher number of patients treated with ECMO.

The discrepancy between the use of VV-ECMO and VV-ECMO for ARDS remains high. Possible explanations include applications, such as for the treatment of acute exacerbation of chronic obstructive pulmonary disease, status asthmaticus, diffuse alveolar haemorrhage, septic shock or bridge to transplant. Nevertheless, it cannot be ruled out that ARDS cases were not coded as such, although they met the Berlin definitions, and were instead improperly coded as pneumonia or acute respiratory insufficiency [23].

### Double-lumen cannula

Double-lumen cannulation is an innovative form of VV-ECMO cannulation that offers a number of advantages, such as a single vascular puncture. However, this work showed that there were few cases in Germany over the last years in ARDS, and the proportion of all ECLS cases involving double-lumen cannulation remained low (9.1%, SD = 1.9%). In comparison, the data from the ELSO Registry showed a notably higher proportion of 25.3% of all ECLS patients with respiratory failure undergoing double-lumen cannulation [3]. This could be due to the type of data documentation in Germany, as the double-lumen cannula has a separate OPS code, but this is only of minor importance in the conversion of fees.

### VA-ECMO

This evaluation showed a massive increase in the use of VA-ECMO from 2012 onwards. How can the sudden increase in VA-ECMO runs be explained? On the one hand, the results of the IABP SCHOCK II Trial 2012 contributed to the abandonment of IABP therapy, leaving clinicians with the need for adequate therapy for critically ill patients with cardiogenic shock, resulting in an increase in VA-ECMO [24]. Another explanation may be the improved availability and easier implementation of ECLS therapy due to the ongoing technical improvements and better biocompatibility of the ECLS devices. One further factor affecting the increase in the number of cases is the possibility of the administration of therapy to patients with post-cardiotomy cardiogenic shock. Another nonmedical cause could be a monetary motivation due to the DRG reimbursement for ECLS therapies. The proportion of VA-ECMO patients with ARDS remained low in 2018 at 11.5%.

### Implications

The results of this work clearly show that the mortality for ECLS therapy in Germany must be improved. The way in which this is achieved, either by instituting a statutory minimum number, mandatory implementation of the existing guidelines or the containment of monetary incentives through a type of accounting according to quality, remains to be answered by further studies.

## Limitations

These are retrospective data. In this case, they were collected in a very structured and representative manner, and since correct data entry affects hospital charges, an increased interest in their correct documentation can be expected. Nevertheless, the informative value of the data is reduced due to possible multiple counting of each patient due to interhospital transfer and the fact that a conversion from VV-ECMO to VA-ECMO may not have been accurately documented. These secondary data provide only case-related in-hospital mortality and cannot provide any information about long-term mortality. Furthermore, there is a lack of precise patient data to verify the indication and detailed information on the time of implantation and the onset of symptoms. Incorrect coding was therefore possible.

## Conclusion

ARDS is a severe disease with a high mortality rate despite ECLS therapy. Although the endpoints and timing of the evaluations differ to those of the CESAR and EOLIA studies and the Extracorporeal Life Support Organization (ELSO) Registry, the reported mortality in these studies was lower than in the present analysis. Further prospective analyses are necessary to evaluate outcomes in ECMO therapy at the centre level.

### Take-home message

Mortality rates for ECLS therapy in Germany remain high and are in contrast to published data in randomized controlled trials and the ELSO Registry.

## Data Availability

The data that support the findings of this study are available from the Federal Statistical Office, but restrictions apply to the availability of these data, which were used under licence for the current study and thus are not publicly available. Data are, however, available from the authors upon reasonable request and with permission of the Federal Statistical Office.

## Abbreviations

ARDS: Acute respiratory distress syndrome
DRG: Diagnosis-related group
E-CPR: Extracorporeal cardiopulmonary resuscitation
ECLS: Extracorporeal life support
ECMO: Extracorporeal membrane oxygenation
ELSO: Extracorporeal life support organization
ECCO_2_R: Pumpless extracorporeal carbon dioxide removal systems
ICD: International statistical classification of diseases and related health problems codes
InEK: Institute for the hospital remuneration system
OPS: Process key
SD: Standard deviation
VA: Venoarterial
VV: Venovenous
VVA: Venovenoarterial
